# First evaluation of a heteroepitaxial diamond ionization chamber operating at low voltage for diagnostic X‐ray dosimetry

**DOI:** 10.1002/mp.70363

**Published:** 2026-02-27

**Authors:** Kiyomitsu Shinsho, Koji Koyama, Keitaro Hitomi, Mitsuhiro Nogami, Osamu Maida, Toshiyuki Onodera, Kanata Kikkawa, Shimma Hashimoto, Yuta Hirai, Yusuke Koba, Ako Haga, Daiki Maruyama, Seong‐Woo Kim

**Affiliations:** ^1^ Graduate School of Human Health Sciences Tokyo Metropolitan University Arakawa‐ku Tokyo Japan; ^2^ Faculty of Health Sciences Tokyo Metropolitan University Arakawa‐ku Tokyo Japan; ^3^ Orbray Co., Ltd. Adachi‐ku Tokyo Japan; ^4^ Graduate School of Engineering Tohoku University Sendai Miyagi Japan; ^5^ Graduate School of Engineering Osaka University Suita Osaka Japan; ^6^ Faculty of Engineering Tohoku Institute of Technology Sendai Miyagi Japan; ^7^ Institute for Radiological Science, National Institutes for Quantum Science and Technology Chiba Japan

**Keywords:** diagnostic X‐ray dosimetry, heteroepitaxial diamond detector, high‐sensitivity dosimetry

## Abstract

**Background:**

Heteroepitaxial diamond has recently gained attention as a radiation detector material due to its wide bandgap, radiation hardness, and near‐tissue equivalence. Despite these advantages, its use as a solid‐state ionization chamber for diagnostic X‐ray dosimetry has not yet been established. Demonstrating stable, high‐efficiency operation at low voltage would enable compact dosimeters with a very small sensitive volume, which is difficult to achieve with conventional air ionization chambers.

**Purpose:**

To perform the first characterization of a heteroepitaxial diamond ionization chamber (HED‐IC) operated at low bias voltage under diagnostic X‐ray conditions and to evaluate its feasibility as a compact, high‐efficiency dosimeter.

**Methods:**

A heteroepitaxial diamond detector (4 × 4 × 0.5 mm^3^) with Ti/Au electrodes was fabricated and evaluated using diagnostic X‐ray beams at tube voltages from 50 to 120 kV. Charge‐collection characteristics, dose linearity, energy dependence, and temporal response were assessed at negative bias voltages with magnitudes between −1 and −100 V. Monte Carlo simulations were performed using PHITS to compute the expected diamond‐to‐air sensitivity ratio under the same beam qualities for comparison with the experimental measurements.

**Results:**

The HED‐IC exhibited excellent dose linearity (*R* ^2^ > 0.997) and weak energy dependence (< 10%) across effective energies from 28.4 to 40.1 keV. The detector enables dose measurements within a very small sensitive volume, only 1/1250 of that of a typical air ionization chamber. The volume‐normalized sensitivity exceeded theoretical expectations, suggesting enhanced effective ionization efficiency. An increased response with higher bias voltage further indicated potential for high‐sensitivity operation.

**Conclusions:**

The results demonstrate that the HED‐IC can operate as a low‐voltage, high‐efficiency solid‐state ionization chamber under diagnostic X‐ray conditions. Owing to the scalability of heteroepitaxial diamond growth, this detector concept provides a promising basis for compact, tissue‐equivalent dosimeters capable of real‐time dose monitoring across a wide range of radiological applications.

## 1 INTRODUCTION

Optimization and management of medical radiation exposure have long been recognized as global priorities. International organizations, including the International Atomic Energy Agency (IAEA) and the World Health Organization (WHO), have emphasized systematic monitoring of patient radiation doses in medical imaging.^1^, ^2^, ^3^ The IAEA has issued international guidelines encouraging digital dose recording and the implementation of Diagnostic Reference Levels (DRLs) for optimization of exposure conditions in clinical practice. In line with these global efforts, several countries—including Japan, which revised its Medical Care Act Enforcement Regulations in 2019—have mandated patient dose recording and management.^4^, ^5^ As a result, the demand for accurate and real‐time dosimetry in diagnostic radiology has grown worldwide, reflecting the global trend toward patient dose optimization.

Detectors used in diagnostic X‐ray dosimetry are required to satisfy several demanding criteria, including high sensitivity to low doses, low energy dependence, small sensitive volume, and real‐time response.^6^, ^7^ Conventional air‐ionization chambers are still widely used in clinical practice and remain the gold standard for dosimetry. Semiconductor detectors such as silicon diodes are compact and sensitive, but they exhibit strong energy dependence, radiation damage effects, and temperature‐dependent response variations, which often necessitate correction factors or frequent recalibration and can compromise measurement reliability in the diagnostic range. Conventional air‐ionization chambers, while highly accurate, also require routine temperature and pressure corrections to ensure dosimetric accuracy under varying environmental conditions. Because the heteroepitaxial diamond ionization chamber (HED‐IC) exhibits very high sensitivity, operation at excessively high bias voltages results in collected charges that exceed the usable or optimal operating range of electrometers commonly used for ionization‐chamber dosimetry; therefore, low‐voltage operation is essential for practical measurements.

Diamond has emerged as a promising detector material because of its wide bandgap (5.5 eV), high carrier mobility, and exceptional radiation hardness. These properties make diamond well suited for use in high‐dose and high‐dose‐rate environments. To date, most studies have focused on single‐crystal chemical vapor deposition (CVD) diamond detectors in the radiation‐therapy energy range.^8^, ^9^, ^10^, ^11^, ^12^, ^13^, ^14^ Commercial detectors such as the PTW microDiamond (T60019, T60023) have demonstrated excellent linearity and reproducibility under megavoltage photon and electron beams. In addition, diamond dosimeters have shown favorable dose‐rate and linear energy transfer (LET) responses under ultra‐high dose‐rate (FLASH) and particle‐beam irradiation conditions.^8^, ^12^, ^15^, ^16^

However, investigations of diamond detectors in the diagnostic X‐ray energy range (20–120 keV) remain extremely limited.^17^, ^18^, ^19^, ^20^ In particular, no previous studies have characterized diamond‐based solid‐state ionization chambers operating in this low‐energy domain. This represents a significant gap in the literature, considering the potential advantages of diamond as a tissue‐equivalent, radiation‐hard, and low‐leakage material suitable for compact, low‐voltage detectors.

Recent advancements in heteroepitaxial diamond growth technology, where non‐diamond substrates such as sapphire or silicon are used, have enabled the fabrication of large‐area, high‐quality diamond wafers through step‐flow growth.^21^, ^22^ This breakthrough enables reproducible device fabrication with minimal variability and offers potential cost advantages compared with single‐crystal CVD diamond, primarily due to its scalability to large‐area wafers and improved manufacturing throughput. Heteroepitaxial diamond thus offers scalability, uniformity, and cost‐effectiveness, making it a strong candidate for next‐generation medical radiation detectors.

In this study, a HED‐IC with dimensions of 4 × 4 × 0.5 mm^3^ was fabricated and evaluated under diagnostic X‐ray conditions. The charge‐collection characteristics, energy dependence, linearity, and stability were investigated under low‐magnitude negative bias voltages (−1 to −50 V). Monte Carlo simulations using PHITS were also performed to analyze the energy dependence of sensitivity and to compare the performance with that of commercially available real‐time dosimeters.

## 2 MATERIALS AND METHODS

### 2.1 Heteroepitaxial diamond sample

**FIGURE 1:**
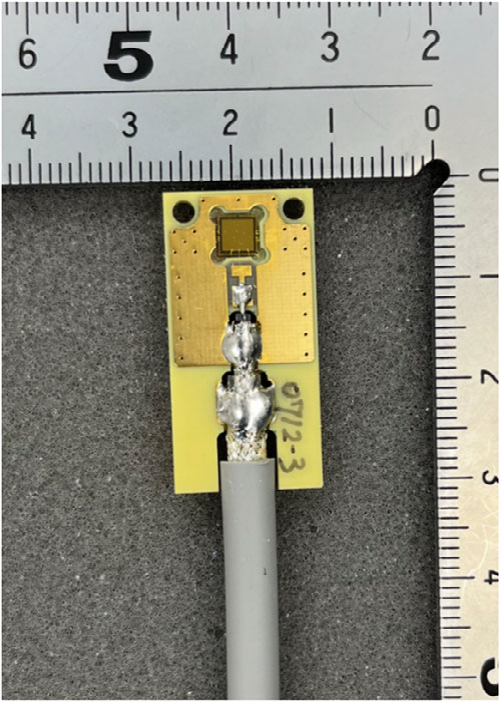
Photograph of the fabricated HED‐IC.

In this study, a heteroepitaxial CVD diamond grown on a sapphire substrate, fabricated by Orbray Co., Ltd. (Japan), was used.^21^, ^22^ The diamond layer was grown by microwave plasma CVD using an iridium buffer layer. The resulting wafer exhibited high crystallinity through step‐flow growth, and a sharp Raman peak was observed at 1332 cm^−^^1^ (the expected first‐order Raman mode of crystalline diamond, commonly used to assess diamond crystal quality), confirming the diamond phase. After removal of the iridium buffer layer and the sapphire substrate, the heteroepitaxial diamond wafer was cut into single‐crystal chips with dimensions of 4 × 4 mm^2^ and a thickness of 0.5 mm using a green‐laser dicing process. Titanium and gold thin films (Ti/Au = 50 nm / 180 nm) were deposited on both surfaces to form electrodes. The Ti layer served to improve the electrical contact between the metal and the diamond surface, while the Au layer ensured electrical stability and chemical durability. Both the top and bottom surfaces were coated with electrodes to construct a double‐sided electrode configuration with a guard ring, which was adopted to ensure a uniform electric‐field distribution, suppress surface leakage currents, and define a well‐controlled active volume. The electrode area without guard ring was 3.1 × 3.1 mm^2^, resulting in an active volume of 3.1 × 3.1 × 0.5 mm^3^. The fabricated detector, referred to as the HED‐IC, was connected to an electrometer (Model EMF523R, EMF Japan Co., Ltd.) to measure the collected charge under applied bias voltages. A photograph of the HED‐IC is shown in Figure 1.

During measurements, the detector was enclosed in a heat‐shrinkable light‐shielding polyethylene covering, because both ultraviolet (UV) and visible light can induce photo‐generated carriers in diamond detectors and increase the measured dark current. This shielding effectively suppresses ambient‐light–induced dark current, allowing stable and reproducible charge measurements under normal clinical lighting conditions.

### 2.2 Experimental setup

All measurements were performed using a diagnostic X‐ray generator (Shimadzu XD150) under a fixed geometry with a source‐to‐chamber distance (SCD) of 100 cm and a field size of 10 × 10 cm^2^. The HED‐IC was positioned on the central X‐ray beam axis. To minimize the effect of backscattered radiation from the patient table, a polystyrene block was inserted between the detector and the table surface. The detector was connected to an electrometer operated in charge‐integration mode with a gate time of 0.5 s. Because the integration mode accumulates both signal and leakage current during exposure, the leakage charge was corrected by subtracting the product of the measured leakage current and the exposure time from the total collected charge. The electrometer automatically recorded both current and integrated charge values at 0.5 s intervals to a connected PC, allowing straightforward determination of the leakage current under beam‐off conditions. The leakage current increased with applied bias voltage; however, because the detector sensitivity also increased with voltage, the relative contribution of leakage charge depended primarily on the delivered dose. Within the experimental conditions of this study, the leakage‐charge contribution ranged from approximately 0.05% to 1.2% of the total collected charge. All measurements were performed with negative bias applied to the bottom electrode (collector electrode) and the top electrode grounded. The reference ionization chamber was calibrated under a standard diagnostic X‐ray beam quality, and energy‐dependent correction factors were applied to account for differences in beam quality for other tube voltages.

### 2.3 Priming (stabilization) protocol

To stabilize the charge‐collection characteristics of the HED‐IC before diagnostic X‐ray measurements, a pre‐irradiation (priming) procedure was performed using a medical linear accelerator (Versa HD, Elekta AB, Sweden). A therapeutic beam was selected because a sufficiently high cumulative dose, defined as that required to reproducibly saturate charge traps and stabilize the charge‐collection characteristics, could not be easily delivered with a conventional diagnostic generator within a reasonable experimental time. Irradiations were conducted with 6 MV X‐rays at a SCD of 100 cm, source‐to‐surface distance (SSD) of 90 cm, and a field size of 10 × 10 cm^2^. The detector was embedded at a depth of 10 cm in a water‐equivalent solid phantom (Tough Water, Kyoto Kagaku) to ensure charged‐particle equilibrium and full scatter conditions, thereby providing a stable and reproducible irradiation environment. To determine an appropriate priming condition, the stabilization behavior was first evaluated by repeatedly irradiating the HED‐IC with 1 Gy per exposure while applying a bias voltage of −5 V to the bottom electrode. The collected charge per 1 Gy was measured after each irradiation to assess the evolution and stabilization of the charge‐collection characteristics. Based on these results, a cumulative dose of 10 Gy was found to be sufficient to achieve a stable operating state.

Accordingly, the priming procedure adopted for all subsequent experiments consisted of X‐ray pre‐irradiation to a total dose of 10 Gy under zero‐bias conditions. This priming step was performed only once as an initial conditioning process and was not required prior to each measurement. Once stabilized, the detector exhibited a stable response under diagnostic X‐ray conditions. Although stabilization of charge‐collection characteristics may also be achieved through other approaches, such as impurity doping during crystal growth or temperature‐based priming, these methods either represent intrinsic material parameters that cannot be modified after device fabrication or introduce additional variables that complicate quantitative control and reproducibility. Although similar stabilization can be achieved through UV irradiation using light‐emitting diodes (LEDs), that method could not be quantitatively controlled. Therefore, X‐ray pre‐irradiation was employed in this study to ensure reproducible and well‐defined priming conditions directly relevant to dosimetric operation.

### 2.4 Dose linearity at low bias

The dose linearity of the HED‐IC under low‐voltage operation was evaluated at five bias voltages: −1, −5, −10, −50, and −100 V. The investigated mAs range was selected to represent entrance skin dose levels typically encountered in general radiography. For each voltage, the detector was irradiated with diagnostic X‐rays while varying the exposure time to deliver different doses. The collected charge was measured at each dose level.

The measurement geometry and setup were identical to those described in Section 2.2, including a diagnostic X‐ray system equipped with a Shimadzu X‐ray tube (0.6/1.2P32ADK‐85, Shimadzu Corporation, Japan), the SCD of 100 cm, and the field size of 10 × 10 cm^2^. Leakage current correction was applied in the same manner as described previously. The applied bias voltage was supplied via the electrometer (EMF523R, EMF Japan). A summary of the measurement conditions is provided in Table 1.

**TABLE 1:** Summary of the measurement conditions.

Parameter	Condition
X‐ray source	Diagnostic X‐ray system equipped with a Shimadzu X‐ray tube (0.6/1.2P32ADK‐85, Japan)
Tube voltage	50 kV, 80 kV, 120 kV
Source‐to‐chamber distance (SCD)	100 cm
Field size	10 × 10 cm^2^
Bias voltage	−1, −5, −10, −50, and −100 V
Measurement mode	Charge integration (0.5 s gate time)
Dose range	0.25–13 mGy
Reference dosimeter	Radcal 10 × 6‐6 ionization chamber

### 2.5 Evaluation of prompt and delayed charge components

The temporal characteristics of charge collection in the HED‐IC were evaluated by separating the induced charge into prompt and delayed components following X‐ray irradiation. Measurements were performed using a diagnostic X‐ray system equipped with a Shimadzu X‐ray tube (0.6/1.2P32ADK‐85, Japan) at tube voltages of 50, 80, and 120 kV and a tube current of 250 mA. For each energy, the irradiation time was varied among 50, 100, 200, 500 ms, and 1 s to investigate the effect of exposure duration on charge‐collection behavior. The experimental geometry and bias configuration were identical to those described in Section 2.2. Immediately after each exposure, the time‐dependent signal was recorded using the electrometer (EMF523R, EMF Japan) in charge‐integration mode.

The prompt component ($Q_{prompt}$) was defined as the charge collected within 1 s after beam‐off, while the delayed component ($Q_{delay}$) corresponded to the charge accumulated between 1 s after irradiation and the time when the signal returned to the dark‐current level. The delayed component is attributed to the gradual release of charge carriers temporarily trapped in the diamond, and therefore reflects trap‐assisted carrier detrapping processes. Each measurement was repeated five times to ensure reproducibility, and the average values were used for analysis. The relationship between ($Q_{prompt}$) and ($Q_{delay}$) as a function of dose was examined to clarify the carrier trapping and release characteristics of the diamond detector.

To more clearly resolve the dependence of charge‐collection dynamics on dose and applied electric field, additional measurements were performed over an extended bias‐voltage range, as discussed in Section 3.3.

### 2.6 Energy dependence

The energy dependence of the HED‐IC was evaluated in the diagnostic X‐ray energy (effective energy: $E_{eff}$) range of 25–45 keV. Measurements were performed using the same geometry described in Section 2.2, at tube voltages of 50, 80, and 120 kV, a tube current of 250 mA, and an exposure time of 200 ms for each condition. A negative bias voltage of −5 V was applied to the bottom (collector) electrode. For each beam quality, the half‐value layer (HVL) was measured in narrow‐beam geometry using aluminum filters, and the corresponding $E_{eff}$ was derived. The collected charge per unit dose, measured using the reference ionization chamber (Radcal 10 × 5‐6 M), was plotted as a function of the effective energy to characterize the X‐ray beam quality.

The Monte Carlo analysis was performed to support the interpretation of the experimental energy‐dependence results by comparing the generated charge in the diamond detector and the reference ionization chamber under the same irradiation conditions. Monte Carlo simulations were conducted using PHITS ver. 3.35.^23^ Based on the geometric shape and material properties, models of both the diamond detector and the reference ionization chamber were implemented, and the absorbed energy in each sensitive volume was calculated under identical beam conditions. The beam energy spectrum was generated using Xcomp5r^24^ based on the X‐ray tube anode angle and HVL information. In the simulation, the beam was irradiated across the entire surface of each detector model, and the energy imparted to the sensitive region was calculated. The absorbed energy was then converted into the expected generated charge using the corresponding *W*‐values (electron–hole pair creation energy ε), with values of 33.97 eV for air and 13 eV for diamond. The results were expressed as the ratio of the diamond detector sensitivity to that of the ionization chamber as a function of effective energy of each beam. A comparison between these two PHITS‐calculated datasets, corresponding to diamond (carbon) and air, was performed to evaluate the theoretical consistency of the energy dependence, focusing on the agreement in the energy‐dependent trend rather than absolute values.

## 3 RESULTS

### 3.1 Priming (stabilization) characteristics

**FIGURE 2:**
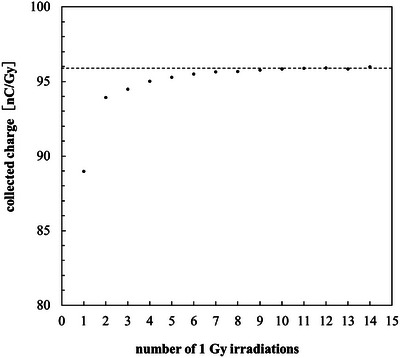
Collected charge per unit dose as a function of the number of 1 Gy irradiations. Bias voltage: −5 V.

Figure 2 shows the collected charge per unit dose (nC/Gy) for successive 1 Gy irradiations. The collected charge increased slightly during the initial exposures and reached a stable level after approximately 12 irradiations. Beyond this point, the average collected charge was 95.91 nC/Gy, with a standard deviation of 0.072 nC/Gy and a coefficient of variation of 0.075 %, indicating excellent stability of charge collection. This stabilization behavior is attributed to the filling of charge traps within the diamond bulk or at the electrode interfaces.

Although X‐ray pre‐irradiation was used for priming in this study, a partial stabilization of the initial charge‐collection behavior has been reported using UV irradiation; however, such UV‐based approaches do not provide bulk stabilization of charge traps.

### 3.2 Dose linearity at low bias

**FIGURE 3:**
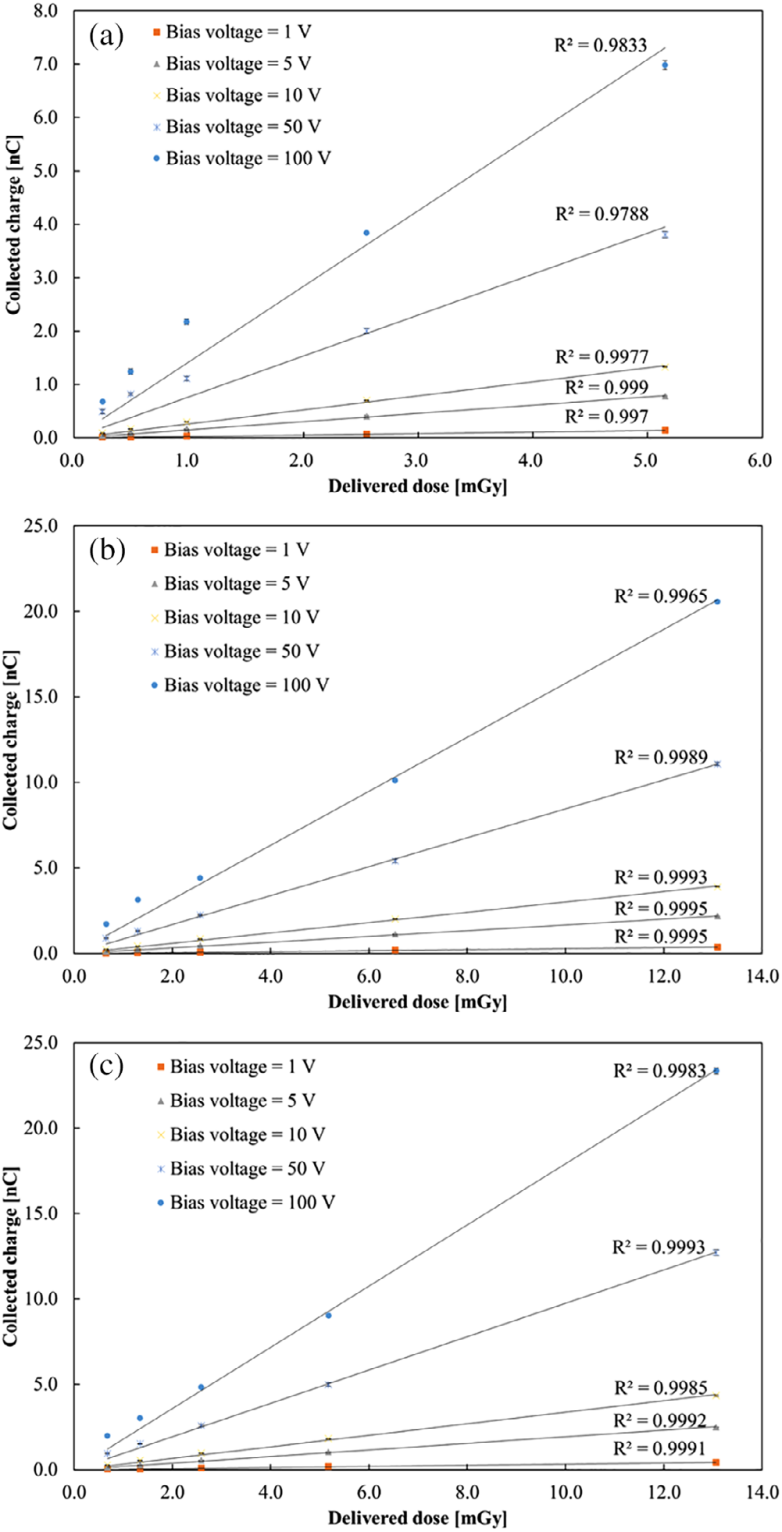
Dose linearity of the HED‐IC at three tube voltages: (a) 50 kV, (b) 80 kV, and (c) 120 kV. Bias voltages: −1 to −100 V (negative bias applied to the bottom electrode).

Figure 3 shows the dose–charge relationships obtained at low bias voltages (1–100 V, negative polarity) for tube voltages of 50 kV (a), 80 kV (b), and 120 kV (c). In all cases, the collected charge increased almost linearly with the delivered dose, indicating a high proportionality even under weak electric field conditions. At 1–50 V, the linear regression coefficients (*R* ^2^) were higher than 0.997, confirming that the HED‐IC maintains good linearity at low bias operation. However, at 100 V, the linearity slightly deteriorated, with *R* ^2^ values somewhat lower than those at moderate bias. At higher doses and higher bias voltages, a slight reduction in the slope of the dose–charge relationship was observed. This behavior can be attributed to space‐charge accumulation and transient polarization under high carrier‐density conditions, which locally reduce the effective charge‐collection efficiency. In addition, at low applied bias voltages where charge collection is incomplete, the collected signal may depend on the spatial distribution of charge generation within the diamond. Because the depth and density of charge generation vary with the X‐ray energy spectrum, spectral differences associated with tube voltage may also contribute to the observed changes in slope, in addition to space‐charge effects. In the present study, the priming procedure was performed without applying a bias voltage. While applying a bias during priming could affect the occupancy of shallow traps and potentially influence subsequent charge‐collection dynamics, the extent to which this contributes to the slight nonlinearity observed at high dose and high bias conditions cannot be quantified from the current dataset and will be investigated in future work.

Overall, the results demonstrate that the HED‐IC provides stable and nearly proportional charge collection in the diagnostic X‐ray dose range even at bias voltages below 100 V, confirming its potential for low‐voltage operation.

**FIGURE 4:**
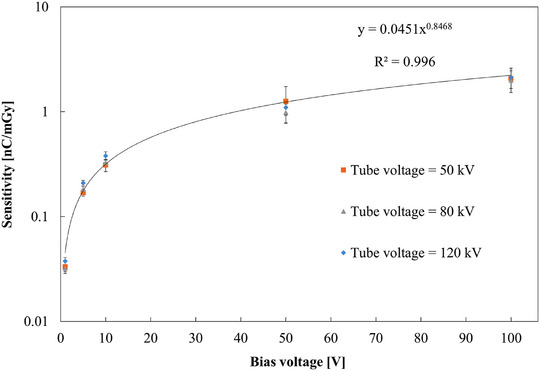
Sensitivity as a function of applied bias voltage for different X‐ray tube voltages. No significant tube‐voltage dependence was observed. The solid line represents a power‐law fit to the data acquired at 120 kV, which were selected as representative. Bias voltages: −1 to −100 V (negative bias applied to the bottom electrode).

In this study, the delivered dose D was evaluated using a calibrated reference air‐filled ionization chamber and was used as a common reference quantity for comparison with the HED‐IC response. Figure 4 shows the dependence of detector sensitivity. The sensitivity (S) was defined as:

$$S=\frac{Q}{D}\;\left[\mathrm{nC}\;\mathrm{mG}y^{-1}\right]$$

where Q is the collected charge and D is the delivered dose determined with a calibrated reference ionization chamber (Radcal 10 × 6‐6). The vertical axis is plotted on a logarithmic scale. In all cases, the sensitivity increased monotonically with increasing bias voltage, showing a sub‐linear dependence on bias voltage. No significant dependence on tube voltage was observed within the examined diagnostic X‐ray range, indicating that the sensitivity is primarily governed by charge‐transport processes in the detector rather than by the X‐ray spectrum. Based on this result, the data acquired at 120 kV were selected as representative, and a power‐law fitting curve was added to Figure 4 to highlight the characteristic voltage dependence. At very low biases (1–10 V), the absolute sensitivity of the HED‐IC was still in the range of 0.05–0.2 nC mGy^−^^1^, i.e. lower than the reference chamber but clearly measurable. At a bias of 50 V, the HED‐IC achieved a sensitivity comparable to that of a conventional air‐filled ionization chamber (Radcal 10 × 6‐6 or 10 × 6‐6 M, ≈ 1 nC mGy^−^^1^). At 100 V, the sensitivity was approximately twice that of the reference chamber. This behavior is physically expected given the much higher material density of diamond compared with air, despite the sensitive volume of the HED‐IC being only 7.22 mm^3^, whereas that of the Radcal 10 × 5‐6 M was approximately 6000 mm^3^.

When normalized to the sensitive volume, the HED‐IC provided an extremely high charge yield per unit volume: about 140‐fold (1 V), 740‐fold (5 V), 1400‐fold (10 V), 4500‐fold (50 V), and 8500‐fold (100 V) higher than the air‐ionization chamber. These values indicate that the high sensitivity of the heteroepitaxial diamond detector originates not from detector volume, but primarily from the high energy deposition associated with the large material density of diamond, together with its efficient charge‐generation and charge‐collection properties. Above 50 V, the increase in sensitivity became less pronounced, suggesting that charge collection approached saturation. The operating bias voltage can be adjusted according to the expected dose range and the charge‐handling capability of the electrometer, providing flexibility in practical measurements.

These findings demonstrate that the HED‐IC can experimentally realize sensitivity equivalent to, or higher than, that of a conventional air‐filled ionization chamber under diagnostic X‐ray conditions at relatively low bias voltages, while offering several‐orders‐of‐magnitude higher volumetric efficiency.

### 3.3 Evaluation of prompt and delayed charge components

**FIGURE 5:**
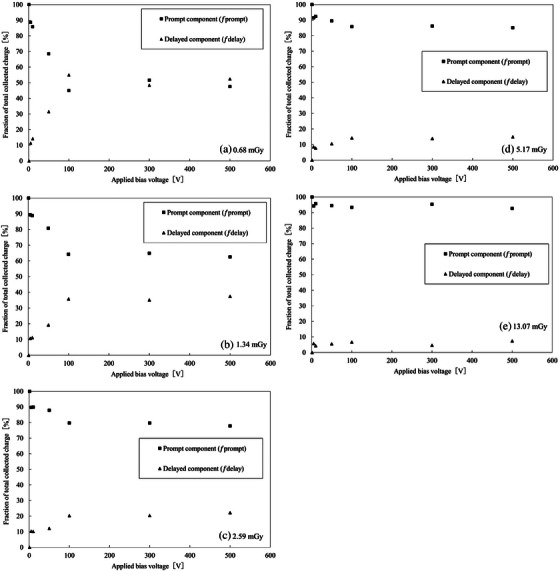
Fractional contributions of prompt and delayed charge components to the total collected charge at X‐ray tube voltage of 120 kV for different delivered doses: (a) 0.68 mGy, (b) 1.34 mGy, (c) 2.59 mGy, (d) 5.17 mGy, and (e) 13.07 mGy. The fractions were defined as $f_{prompt}=Q_{prompt}\;/Q_{total}$ and $f_{delay}=Q_{delay}\;/Q_{total}$, where $Q_{total}=Q_{prompt}+Q_{delay}$. The prompt and delayed components are complementary quantities normalized to 100%, and therefore a common y‐axis range of 0%–100% is used for all panels to facilitate direct comparison across bias voltages and dose levels. Bias voltages ranged from −1 to −500 V, with negative bias applied to the bottom electrode.

Figure 5 shows the fractional contributions of the prompt and delayed charge components, where the delayed component originates from trap‐assisted carrier release following X‐ray irradiation, normalized to the total collected charge (defined as 100%), as a function of applied bias voltage at an X‐ray tube voltage of 120 kV. Five dose levels (0.68–13.07 mGy) were analyzed to investigate the effect of exposure on charge‐collection dynamics. Each data point represents the mean of five repeated measurements. The experimental configuration and signal integration procedure followed those described in Section 2.5.

At the lowest dose (0.68 mGy; Figure 5A), the prompt component dominated at low bias voltages, whereas the fractional contribution of the delayed component increased with increasing bias voltage. This behavior is consistent with the interpretation that under weak‐field conditions, most charge carriers were rapidly collected, but as the electric field strengthened, additional carriers trapped in shallow levels were gradually released and contributed to the delayed signal. As the delivered dose increased, the dependence on bias voltage became less pronounced. At the highest dose (13.07 mGy; Figure 5E), the prompt component was dominant even at low voltages, and the fraction remained almost constant with bias. This indicates that at high carrier densities the available traps were partially saturated, leading to a faster overall charge‐collection process. At low doses, where the total collected charge is small, the contribution from trap‐assisted delayed charge release becomes relatively significant compared to the prompt component, whereas at higher doses this contribution becomes negligible due to partial trap filling.

These results indicate that the charge‐collection dynamics in the HED‐IC depend not only on the applied electric field but also on the carrier generation density determined by the X‐ray dose. The combined dependence on bias voltage and dose can be interpreted phenomenologically as reflecting competition between carrier drift, trapping, and recombination processes within the heteroepitaxial diamond layer.

### 3.4 Energy dependence

**FIGURE 6:**
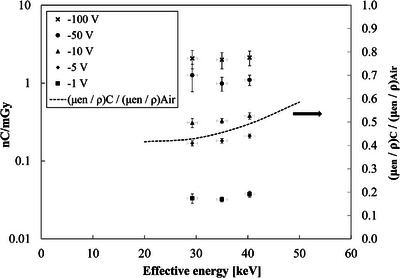
Sensitivity (nC/mGy) of the heteroepitaxial diamond ionization chamber (HED‐IC) as a function of effective X‐ray energy for different negative bias voltages (−1 to −100 V, applied to the bottom electrode). The mass energy‐absorption coefficient ratio of carbon (representing diamond) to air (C/air) derived from the NIST XCOM database^25^ is also shown as a reference using a secondary y‐axis.

Figure 6 shows the sensitivity of the HED‐IC as a function of effective X‐ray energy for bias voltages of 1, 5, 10, 50, and 100 V (−1 to −100 V applied to the bottom electrode). At all bias voltages, the sensitivity increased slightly with effective energy from 28.4 to 40.1 keV. The overall variation, expressed as the ratio of maximum to minimum sensitivity within this range, was 6.8% (1 V), 9.0% (5 V), 8.8% (10 V), 10.0% (50 V), and 2.7% (100 V). No clear correlation between applied bias voltage and energy dependence was observed.

These results indicate that, even without any energy‐compensation filter, the HED‐IC exhibits an energy dependence within approximately 10% over the diagnostic X‐ray range. Such behavior demonstrates that the detector response is only weakly dependent on photon energy under practical operating conditions.

## 4 DISCUSSIONS

### 4.1 Energy dependence

**FIGURE 7:**
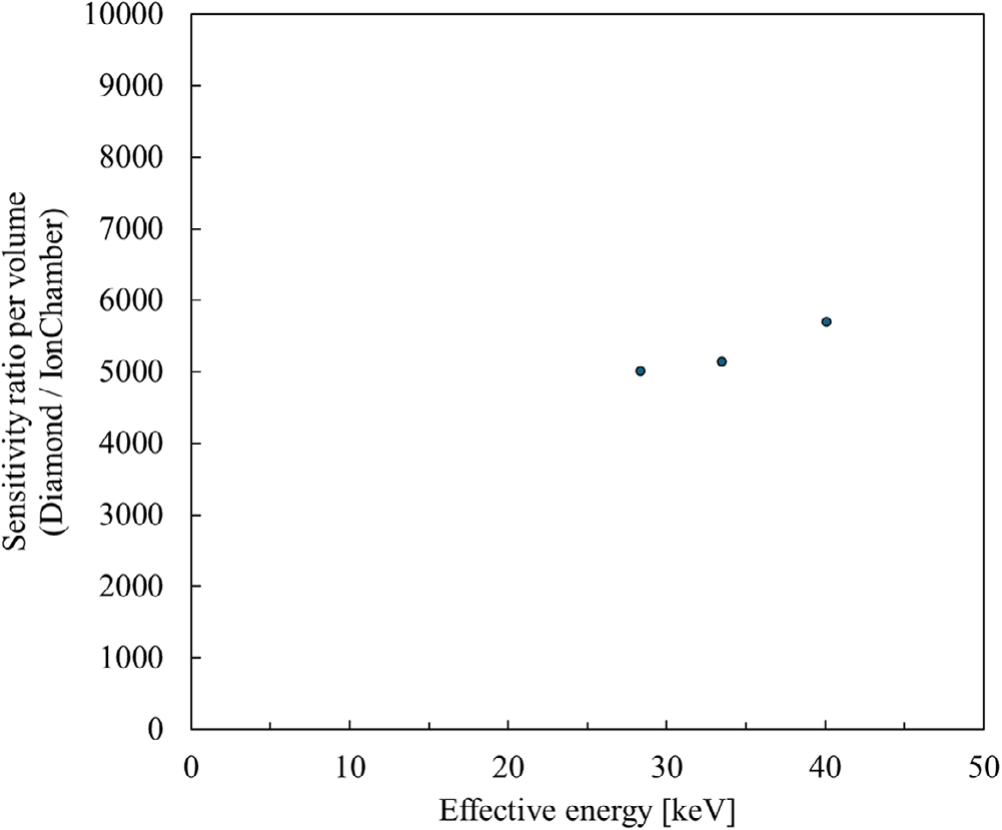
PHITS‐calculated volume‐normalized sensitivity ratio (diamond/ion chamber) as a function of effective X‐ray energy in the diagnostic range (28.4–40.1 keV). This ratio represents the relative absorbed energy per unit sensitive volume between diamond and the reference air‐filled ionization chamber.

Figure 7 shows the PHITS‐calculated volume‐normalized sensitivity ratio (diamond/air‐ionization chamber) as a function of effective X‐ray energy in the diagnostic range (28.4–40.1 keV). In this calculation, the sensitivity ratio was derived from the ratio of generated charge in each sensitive volume, taking into account the average energy required to produce one ion pair (*W*‐value). The *W*‐values used were 33.97 eV^26^ for air and 13 eV^27^ for diamond. Thus, the calculated ratio represents a theoretical charge‐generation efficiency that reflects both photon energy absorption and ionization yield characteristics of each material. The calculated sensitivity ratio was approximately 5 × 10^3^–6 × 10^3^ within the photon energy range of 28.4–40.1 keV and exhibited a weak upward trend with increasing energy. This magnitude is primarily governed by the large difference in material density and *W*‐value between diamond and air. The observed weak energy dependence reflects secondary effects, including gradual changes in the dominant photon‐interaction processes and the mass energy‐absorption coefficient ratio of carbon (representing diamond) to air (C/air). Based on NIST XCOM data.^25^ C/air increases moderately from approximately 0.43 at 28 keV to approximately 0.49 at 40 keV, contributing to the observed gradual trend while remaining a secondary factor.

Experimentally, as shown in Figure 6, the HED‐IC also exhibited minimal energy dependence, with the sensitivity variation remaining within approximately 2.7%–10% across 28.4–40.1 keV. In this figure, the calculated C/air curve derived from NIST XCOM data^25^ is included as a reference using a secondary y‐axis to facilitate direct comparison. Importantly, the measured trend at low bias voltages (1–10 V, negative polarity) was in good agreement with the PHITS calculation, indicating that even under weak electric‐field conditions, charge collection is sufficiently stable and that the energy dependence is primarily governed by photon–material interaction processes. At 100 V, the energy dependence became slightly smaller, which may be related to a more reproducible detector response after pre‐irradiation (priming). Although the detailed mechanism is not fully understood, priming may help establish a reproducible initial state of charge trapping in the diamond. Minor fluctuations in field strength can nevertheless occur over time due to temperature changes or accumulated usage history, leading to small variations in measured sensitivity. Consequently, no significant correlation between applied bias voltage and energy dependence was observed.

Overall, both experimental and simulation results consistently demonstrate that the HED‐IC exhibits very weak energy dependence in the diagnostic X‐ray range investigated in this study (28.4–40.1 keV). These findings indicate that the HED‐IC provides a practically flat response in this range and may reduce or potentially eliminate the need for complex energy‐compensation filters, supporting its suitability as a stable and high‐sensitivity dosimeter for diagnostic radiology.

### 4.2 Sensitivity ratio to an ionization chamber

Table 2 summarizes the comparison of both absolute and volume‐normalized sensitivities between the HED‐IC and representative commercial dosimeters. The volume‐normalized sensitivity ratio (Diamond/Air) of the HED‐IC obtained by PHITS simulations (Figure 7) generally agrees with the theoretical trend in the low‐voltage region, while it exceeds the theoretical range at −50 V and becomes even higher at −100 V. Because the PHITS calculation represents a theoretical indicator based on the ratio of charge generation within the sensitive volume (*W*‐value: 33.97 eV for air and 13 eV for diamond), the experimental sensitivity exceeding the theoretical prediction suggests that the effective ionization generation efficiency is higher than that expected from the nominal *W*‐value. A similar tendency is observed when the HED‐IC is compared with other diamond‐based detectors, as described below.

**TABLE 2:** Comparison of absolute sensitivities and volume‐normalized sensitivities between the HED‐IC and representative reference dosimeters. The values for volume‐normalized sensitivity (nC mGy^−^^1^ mm^−^^3^) allow direct comparison of detector response per unit sensitive volume. The volume‐normalized sensitivity ratio is defined as the ratio of the volume‐normalized sensitivity (sensitivity divided by active volume) of each detector to that of the Radcal 9015 (10 × 6‐6) ionization chamber, which is taken as unity.

Detector	Active volume (mm^3^)	Sensitivity (nC mGy^−1^)	Sensitivity ratio	Volume‐normalized sensitivity ratio (relative to Radcal 9015)
Radcal 9015 10 × 6‐6^28^	6000	2.1 × 10^−1^	1	1
PTW 30013 farmer chamber^29^	600	2.0 × 10^−2^	9.5 × 10^−2^	9.5 × 10^−1^
IBA dosimetry CC13^30^	130	3.6 × 10^−3^	1.7 × 10^−2^	7.9 × 10^−1^
PTW 60019 microdiamond^31^	0.004	1 × 10^−3^	4.8 × 10^−3^	7.1
PTW microSilicon T60023^31^	0.03	1.9 × 10^−2^	9.1 × 10^−2^	1.8 × 10^1^
HED‐IC −1 V	4.80	3.4 × 10^−2^	1.6 × 10^−1^	2.0 × 10^2^
HED‐IC −5 V	4.80	1.9 × 10^−1^	9.0 × 10^−1^	1.1 × 10^3^
HED‐IC −10 V	4.80	3.4 × 10^−1^	1.6	2.0 × 10^3^
HED‐IC −50 V	4.80	1.1	5.4	6.5 × 10^3^
HED‐IC −100 V	4.80	2.2	1.0 × 10^1^	1.3 × 10^4^

The PTW 60019 microDiamond operates without an applied bias, and its corrected volume‐normalized sensitivity is 7.1 nC mGy^−^^1^ mm^−^^3^.^31^ This value is substantially lower than the sensitivity measured for the HED‐IC even at the lowest applied bias of −1 V (2.0 × 10^2^ nC mGy^−^^1^ mm^−^^3^), and the difference becomes progressively larger at higher voltages. The exceptionally high volumetric sensitivity achieved by the HED‐IC therefore cannot be explained solely by the intrinsic *W*‐value of diamond. Instead, it suggests that additional implementation‐dependent factors—such as interfacial electric fields, trap‐related carrier dynamics, and geometrical scaling effects—significantly enhance the effective ionization yield in the HED‐IC. These possible mechanisms are discussed in the following sections.

1. Influence of interfacial effects
  The relative importance of interfacial effects strongly depends on the surface‐to‐volume ratio of the sensitive volume. Interfacial electric fields and surface states at the electrode–diamond boundary can affect charge generation and carrier transport in diamond detectors, particularly in devices with extremely small sensitive volumes. In the PTW 60019 microDiamond, the very large surface‐to‐volume ratio, which is on the order of 10^2^–10^3^ mm^−^^1^ based on the manufacturer‐reported geometry, allows such interfacial contributions to become relatively significant.
2. Electric‐field–enhanced bulk charge‐collection in the HED‐IC
  Unlike the microDiamond, the HED‐IC operates under an externally applied electric field. This field substantially enhances carrier drift and suppresses recombination losses within the diamond bulk. As the applied voltage increases, the charge‐collection efficiency improves markedly, leading to a pronounced increase in the volume‐normalized sensitivity. These electric‐field–dependent bulk effects, together with implementation‐specific factors such as electrode configuration and device geometry, provide a plausible explanation for the exceptionally high sensitivity observed in the HED‐IC.

Although the contribution of interfacial effects in the HED‐IC is expected to be smaller than in the microDiamond, the influence of the electrode–diamond interface on charge generation and transport cannot be entirely neglected and may play a modest role in enhancing the detector response. In addition, bulk defects and impurity‐related levels, i.e., localized states within the band gap associated with impurities and defect complexes intrinsic to heteroepitaxial diamond, may further modify the charge‐generation and collection processes, potentially increasing the effective ionization yield beyond that predicted from the nominal *W*‐value of diamond (13 eV).

The combined influence of these factors allows the HED‐IC to achieve high sensitivity while maintaining near–tissue equivalence, making it a promising low‐voltage, high‐efficiency dosimeter for diagnostic X‐ray applications. Future work aimed at quantitatively elucidating how interfacial effects, bulk defects, and electric‐field–enhanced collection contribute to the effective *W*‐value will be essential for improving the accuracy, reliability, and absolute dosimetric performance of diamond‐based ionization chambers.

### 4.3 Overall interpretation

When operated at low bias voltages, the HED‐IC demonstrated a sensitivity comparable to that of a conventional ionization chamber, despite having only 1/1250 of the sensitive volume. The effective temporal response depends on the applied bias voltage, dose level, and measurement conditions, reflecting varying contributions of prompt charge collection and delayed, trap‐assisted charge release. This indicates that both low‐ and high‐dose measurements can be achieved within a very small sensitive volume, while near‐real‐time dose monitoring is achievable particularly at moderate to high dose levels across a wide dynamic range.

At higher applied voltages, the HED‐IC exhibited even greater sensitivity, highlighting its potential as a highly sensitive, tissue‐equivalent detector suitable for medical applications. Although the present evaluation was performed using a small‐area prototype (4 × 4 mm^2^), heteroepitaxial diamond substrates with dimensions up to 50 × 50 mm^2^ have already been demonstrated.^21^, ^22^ By applying the same device concept to such large‐area wafers, the achievable sensitivity is expected to increase by more than two orders of magnitude compared with typical ionization chambers, potentially enabling precise dosimetry in regions that have previously been difficult to measure. Further improvements in diamond crystal quality and electrode–diamond interface engineering are expected to enhance charge‐collection speed and reduce fluctuations in future devices.

These improvements in sensitivity were achieved while maintaining the inherent tissue equivalence of diamond, supporting its applicability as a reliable dosimetric material across a wide range of clinical conditions.

These characteristics imply that the HED‐IC has strong versatility and scalability, offering applicability across a wide range of clinical and research settings—from diagnostic radiology to therapeutic radiation dosimetry. Ultimately, a single HED‐IC device could serve as a unified detector covering both low‐ and high‐dose domains, bridging the gap between real‐time monitoring and high‐sensitivity cumulative dose measurement.

## 5 CONCLUSIONS

This study presented the first evaluation of a HED‐IC operating at low voltage for diagnostic X‐ray dosimetry. The HED‐IC demonstrated stable and nearly proportional charge collection even at bias voltages down to −100 V, achieving sensitivity comparable to that of a conventional air‐ionization chamber despite having only 1/1250 of its sensitive volume. The detector also exhibited very weak energy dependence (within 10%) and excellent temporal stability, confirming its suitability for real‐time dosimetry in the diagnostic range.

The experimental results, supported by PHITS simulations, revealed that the high volumetric efficiency of the HED‐IC arises from a combination of diamond's intrinsic charge‐generation properties and electric‐field–enhanced bulk charge collection, together with implementation‐dependent factors such as device geometry and electrode configuration. At higher bias voltages, the sensitivity exceeded that of conventional chambers, highlighting its potential as a highly sensitive, tissue‐equivalent dosimeter. Although the present evaluation was performed using a small‐area prototype (4 × 4 mm^2^), the scalability of heteroepitaxial diamond growth suggests that larger‐area devices could be realized in future, potentially expanding applicability to a broader dose range.

These findings demonstrate that the HED‐IC combines the advantages of solid‐state and ionization‐chamber detectors, providing real‐time, high‐resolution, and wide‐dynamic‐range dosimetry capabilities. With further optimization of electrode design and geometry, the HED‐IC concept could evolve into a versatile detector platform applicable from diagnostic radiology to therapeutic radiation dosimetry.

## CONFLICT OF INTEREST STATEMENT

Koji Koyama and Seongwoo Kim are employees of Orbray Co., Ltd. The remaining authors declare no conflicts of interest.

## Data Availability

The data that support the findings of this study are available from the corresponding author upon reasonable request.
